# Direct Oral Anticoagulants in the Cardiorenal Patient: Pharmacology, Kidney Function Estimation and Dose Selection Across the CKD Spectrum

**DOI:** 10.3390/cimb48101085

**Published:** 2026-10-20

**Authors:** Alessandra Sorce, Maria Elena Ciuppa, Roberta Germanà, Giancarlo Altieri, Claudio Zinno, Carmelo Zumbino, Giuseppe Puccia, Salvatore Evola, Giuseppe Mulè, Caterina Carollo

**Affiliations:** 1 Department of Health Promotion, Mother and Child Care, Internal and Specialistic Medicine, University of Palermo, 90127 Palermo, Italy; alessandra.sorce@community.unipa.it (A.S.); mariaelena.ciuppa@community.unipa.it (M.E.C.); roberta.germana@community.unipa.it (R.G.); giancarlo.altieri@community.unipa.it (G.A.); claudio.zinno@community.unipa.it (C.Z.); giuseppe.mule@unipa.it (G.M.); 2 Department of Emergency, “Paolo Giaccone” University Hospital, 90100 Palermo, Italy; carmelo.zumbino@policlinico.pa.it; 3 Division of Cardiology, “Paolo Giaccone” University Hospital, 90100 Palermo, Italy; giuseppe.puccia@asp.rg.it; 4 Catheterization Laboratory, Department of Medicine and Cardiology, Azienda Ospedaliera Universitaria Policlinico “P. Giaccone”, 90127 Palermo, Italy; salvatore.evola@policlinico.pa.it

**Keywords:** direct oral anticoagulants, chronic kidney disease, atrial fibrillation, cardiorenal syndrome, renocardiac syndrome, glomerular filtration rate, Cockcroft–Gault, CKD-EPI, anticoagulant-related nephropathy

## Abstract

Atrial fibrillation (AF) and chronic kidney disease (CKD) are not two coincident diseases but two expressions of the cardiorenal syndrome, the disorder in which dysfunction of the heart or the kidney induces dysfunction of the other. Their coexistence spans the chronic renocardiac and cardiorenal subtypes of that syndrome, and fits neither cleanly: the relationship is genuinely bidirectional, and each organ accelerates the deterioration of the other. Anticoagulation sits precisely at the point where this bidirectional relationship becomes a therapeutic dilemma: the same patient is simultaneously hypercoagulable and haemorrhagically fragile, and the drug intended to protect the brain is cleared by an organ whose function is both impaired and unstable. Direct oral anticoagulants (DOACs) have redefined therapeutic standards in AF and venous thromboembolism (VTE), offering predictable pharmacokinetics, fewer food and drug interactions and reduced monitoring requirements compared with vitamin K antagonists (VKAs). In the cardiorenal patient, however, these advantages are conditional. Renal elimination varies more than three-fold across the class—from approximately 27% for apixaban to approximately 80% for dabigatran—so that the choice of molecule, and not merely the decision to anticoagulate, becomes a kidney-related decision. The cardiorenal argument in favour of DOACs extends beyond bleeding: VKAs promote vascular calcification through inhibition of matrix Gla protein, are associated with calciphylaxis, and can cause anticoagulant-related nephropathy, an under-recognised cause of accelerated loss of glomerular filtration rate (GFR). Randomised and observational data indicate that GFR declines faster on VKAs than on DOACs, making the kidney an outcome of anticoagulation and not only a route of elimination. A second, largely unresolved problem is metrological rather than pharmacological. Dose thresholds for DOACs were derived from creatinine clearance (CrCl) estimated with the Cockcroft–Gault (CG) equation, whereas contemporary nephrology stages kidney disease with estimated GFR (eGFR) from the CKD-EPI or EKFC equations. The two quantities are not interchangeable, and the discordance between them translates directly into discordant doses in 4–34% of patients depending on the agent, with the largest discrepancies in the oldest patients and in those with the lowest measured function. This review reframes DOAC therapy as a cardiorenal problem. We examine the shared pathophysiological substrate of AF and CKD, the pharmacology of each agent through the lens of renal handling, the evidence across CKD stages G1–G5D including dialysis, the kidney as an outcome rather than only a covariate of anticoagulation, and the clinical consequences of the equation used to estimate kidney function at the point of prescription.

## 1. Introduction

For more than a century, anticoagulation has been central to the management of conditions carrying a high thrombotic risk, principally AF and VTE [1,2]. In routine care, DOACs are also associated with lower rates of recurrent VTE, hospitalisation and death than warfarin [3]. The discovery of heparin and the subsequent introduction of VKAs such as warfarin transformed the treatment of thromboembolic disease, but the intrinsic limitations of VKAs—a narrow therapeutic window, the need for frequent international normalised ratio (INR) monitoring, and numerous drug and food interactions [4]—drove the development of more manageable agents [5]. DOACs act selectively on single steps of the coagulation cascade: apixaban, edoxaban and rivaroxaban directly inhibit activated factor X (FXa), whereas dabigatran directly inhibits thrombin (activated factor II, FIIa) [6]. Their clinical advantages—rapid onset, predictable exposure, no routine monitoring and a consistently lower risk of intracranial haemorrhage than VKAs—follow directly from pharmacokinetic and pharmacodynamic profiles far more predictable than those of VKAs [7], and are now well established [8]. Meta-analyses of the pivotal trials have quantified the benefit separately for stroke prevention in non-valvular AF [9] and for the treatment of VTE [10].

What this class-level account omits is that a large and growing share of anticoagulated patients do not have normal kidney function. CKD affects an increasing proportion of the population, particularly older people and those with diabetes, hypertension and cardiovascular disease [11], and these are the same patients in whom AF is most prevalent [12]. The relationship is bidirectional and self-reinforcing: reduced GFR promotes atrial remodelling and arrhythmogenesis, while AF is itself associated with incident kidney disease and faster GFR decline [13,14].

This is the territory of the cardiorenal syndrome, defined as a disorder of the heart and kidneys in which acute or chronic dysfunction of one organ induces acute or chronic dysfunction of the other, and divided by the Acute Dialysis Quality Initiative into five subtypes according to the primary organ and the time course [15,16]. The coexistence of AF and CKD sits across two of them. Where kidney disease comes first, the chronic renocardiac subtype (type 4) describes how uraemia, disordered mineral metabolism, chronic inflammation and haemodynamic overload produce atrial fibrosis, left ventricular hypertrophy and an arrhythmogenic substrate. Where the cardiac abnormality comes first, the chronic cardiorenal subtype (type 2) describes how it accelerates loss of kidney function.

It should be said plainly that AF fits this framework imperfectly. The classification was constructed around heart failure and around an identifiable temporal sequence, and in the patient who presents with both AF and CKD the sequence is usually unknowable and arguably irrelevant, since the two conditions share pathways—fibrosis, inflammation, endothelial dysfunction, neurohormonal activation—rather than standing in a simple causal line [17]. The working group that produced the classification acknowledged that patients move between subtypes and that it was never intended to assign each patient to one category. For the purposes of this review, the useful element of the framework is not the subtype label but its founding premise: that the two organs must be treated as one system. Treating CKD as a dosing covariate is precisely the error the concept was formulated to prevent.

This distinction has practical consequences. In a patient with CKD, the decision to anticoagulate involves at least four kidney-dependent judgements that a purely cardiological framing tends to collapse into one: (i) whether the thromboembolic benefit outweighs a bleeding risk that rises steeply and non-linearly as GFR falls; (ii) which molecule to use, given that renal clearance differs three-fold across the class [18]; (iii) what dose to use, given that the labelled thresholds are expressed in a unit (CG-estimated CrCl) that is not the unit used to stage the patient’s kidney disease [19]; and (iv) how the chosen drug will itself affect the kidney and the vasculature over time [20,21].

Current European guidance recommends DOACs over VKAs for stroke prevention in AF, including in most degrees of kidney impairment, while leaving the dialysis population explicitly unresolved [22]. Registration trials of DOACs used the CG equation to estimate CrCl and to guide dose reduction [23]. The CKD-EPI equation provides a more accurate estimate of GFR and a better classification of kidney disease, but it was not the equation used to generate the dosing evidence [24]. It is important to be explicit about what this means: CG is not a “gold standard” for measuring kidney function—it is a regulatory reference for dose selection, and its persistence reflects the provenance of the dosing evidence rather than its metrological superiority [19,24]. Substituting a more accurate GFR equation may in principle improve dose selection in frail patients or those with borderline function [25], but it also shifts patients across dosing thresholds in ways that have not been prospectively validated against clinical outcomes [26].

This review therefore has three aims: to describe the cardiorenal substrate that makes anticoagulation in CKD distinctive; to compare DOACs with each other and with VKAs across the CKD spectrum with explicit attention to kidney outcomes and not only bleeding; and to analyse the clinical consequences of the equation chosen to estimate kidney function at the point of prescription.

## 2. Materials and Methods

This is a narrative review. Its aim is an interpretive synthesis of the literature on DOACs and VKAs in CKD, and on the equations used to estimate CrCl and eGFR for dose selection, rather than an exhaustive or protocol-driven retrieval of the evidence.

PubMed and Google Scholar were searched between June and August 2026, using combinations of the terms “DOAC”, “NOAC”, “VKA”, “warfarin”, “chronic kidney disease”, “cardiorenal”, “atrial fibrillation”, “Cockcroft–Gault”, “CKD-EPI”, “eGFR” and “creatinine clearance”. The search was limited to reports published in English. No restriction was placed on year of publication: the sources cited span 1999 to 2025, with about half published from 2020 onwards, reflecting a field in which the pivotal trials date from 2009 to 2013 while the evidence on kidney outcomes and on estimating equations is largely recent. Titles and abstracts were screened by three of the authors (CC, AS, MC), and the reference lists of the retrieved articles and of relevant reviews were examined for further sources.

Selection was purposive rather than exhaustive. Priority was given to randomised trials and their pre-specified renal analyses, to cohort studies addressing kidney outcomes on anticoagulation, to pharmacokinetic studies in kidney impairment and dialysis, and to the studies that compare dose selection across estimating equations, which form the core of Section 7. Current European guidance and regulatory labelling were consulted directly for dosing thresholds. Case reports and small series were used only where no larger evidence exists, as for anticoagulant-related nephropathy. In keeping with the narrative design, no formal risk-of-bias assessment, quantitative synthesis or PRISMA-guided flow of records was undertaken, and the strength of the evidence is instead discussed alongside each claim in the text.

## 3. The Cardiorenal Substrate: Why Anticoagulation in CKD Is a Different Problem

### 3.1. A Bidirectional Epidemiology

AF is common in the general population but substantially more so in kidney disease. In patients with CKD stage G3 or worse, reported prevalence reaches 25–30%, and in dialysis populations it exceeds 30%, with values above 40% in older and frail individuals [27,28]. The traffic runs in both directions: incident AF is associated with a higher risk of progression to end-stage kidney disease [13], and AF is independently associated with kidney disease, cardiovascular events and death across cohorts [14]. CKD, in turn, is an independent predictor of incident AF and of poor survival thereafter [29].

When the two coexist, thromboembolic risk increases more than the sum of its parts [30]. Advanced CKD (stages G4–G5) is recognised as an independent predictor of adverse cardiovascular and cerebrovascular events and of mortality [13,30]. Crucially, the same patients are also more prone to haemorrhage, because of uraemic platelet dysfunction, vascular fragility and accumulation of renally cleared drugs [31]. Observational and trial data consistently show that patients with both AF and CKD experience more ischaemic stroke and more intracranial bleeding than patients with preserved kidney function [32].

The pathogenesis of AF in CKD is multifactorial and largely cardiorenal in character: chronic hypertension and haemodynamic overload, left ventricular hypertrophy and atrial stretch, myocardial and atrial fibrosis, systemic inflammation, oxidative stress, disordered mineral metabolism and electrolyte shifts—including those imposed intermittently by haemodialysis—together create and maintain the arrhythmogenic substrate [28].

### 3.2. The Uraemic Coagulopathy: Hypercoagulable and Haemorrhagic at Once

CKD produces a paradoxical haemostatic state. On the prothrombotic side, plasma levels of fibrinogen, factor VIII and von Willebrand factor are increased, while the activity of physiological anticoagulant mechanisms such as antithrombin III and protein C is reduced; chronic inflammation and endothelial dysfunction amplify thrombus formation [33]. On the haemorrhagic side, uraemic toxins impair platelet adhesion and aggregation, anaemia alters rheological margination of platelets, and vascular wall integrity is compromised [31,34].

This duality is the defining feature of the cardiorenal anticoagulation problem, and it explains why risk scores derived in populations with preserved kidney function transfer poorly to CKD: the same patient occupies a high decile of both the thrombotic and the haemorrhagic distribution, and the two risks are driven by shared rather than independent mechanisms.

### 3.3. The Cardiorenal Cost of Vitamin K Antagonism

VKAs inhibit vitamin K epoxide reductase, preventing γ-carboxylation of the vitamin K-dependent factors II, VII, IX and X and of proteins C and S. The same γ-carboxylation is required to activate matrix Gla protein, the principal local inhibitor of vascular calcification. VKA therapy has therefore been associated with progression of vascular and valvular calcification and, rarely but severely, with calciphylaxis—complications of particular relevance in a dialysis population already burdened by disordered mineral metabolism [35,36]. The causal direction is supported from the opposite side: in the randomised VitaVasK trial, vitamin K1 supplementation in haemodialysis patients reduced 18-month progression of thoracic aortic calcification by an average of 56% relative to standard care, with a concordant trend for coronary calcification and a 69% fall in circulating uncarboxylated matrix Gla protein [37]. If restoring vitamin K activity slows calcification, antagonising it plausibly accelerates the same process—which is the mechanistic core of the cardiorenal case against VKAs, and one that no bleeding endpoint captures.

VKAs are also pharmacologically less predictable as GFR falls. Kidney function influences warfarin dose requirement, time in therapeutic range and haemorrhagic complications [38], and major haemorrhage is frequent in the first year of therapy in elderly patients with AF [39], and in advanced CKD the net clinical benefit of warfarin for stroke prevention becomes genuinely uncertain [40,41]. Observational data in CKD cohorts have reported worse outcomes with warfarin in this setting [42,43].

### 3.4. Anticoagulant-Related Nephropathy: The Kidney as a Target of Therapy

The best-characterised example of anticoagulation acting on the kidney rather than merely being handled by it is warfarin-related nephropathy (WRN) [20,44]. WRN typically follows over-anticoagulation, classically at an INR above 3.0. Excessive hypocoagulability permits glomerular haemorrhage, with passage of erythrocytes into the tubular lumen, formation of occlusive red cell casts and secondary acute tubular injury [44]. The result is an abrupt and often silent decline in kidney function that is easily misattributed to the natural progression of CKD.

Diagnosis is difficult. WRN has no specific symptom and no reliable urinary signature; the usual signal is an unexplained rise in serum creatinine, sometimes with microscopic haematuria. Biopsy confirmation is frequently contraindicated in an anticoagulated patient, so the condition is probably under-recognised [20]. It is not benign: WRN has been associated with increased renal and cardiovascular mortality and with acceleration towards dialysis, and it occurs preferentially in patients who already have CKD stage G3 or worse [20,44].

**Figure 1:**
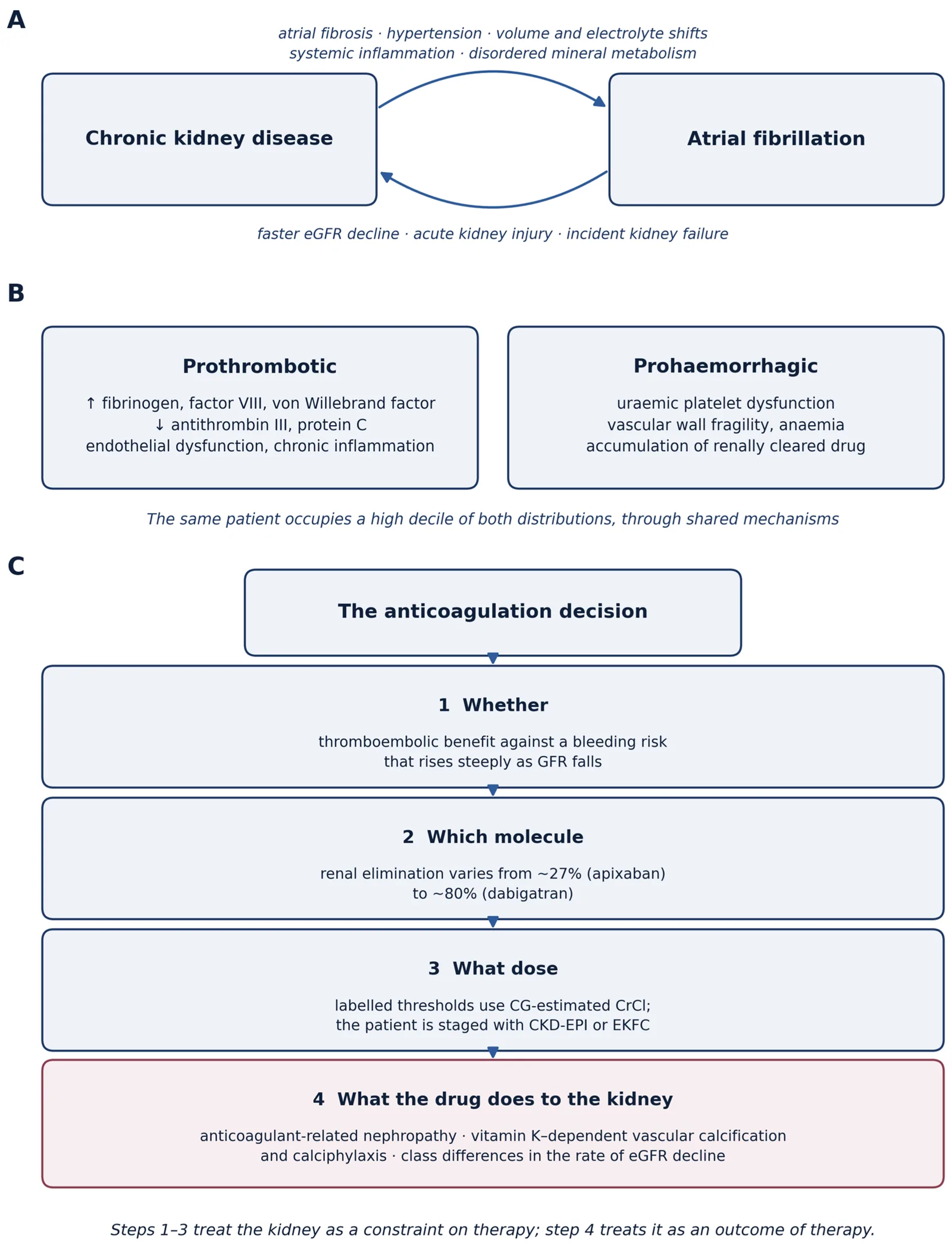
The cardiorenal continuum and the anticoagulation decision. (**A**) Atrial fibrillation and chronic kidney disease reinforce one another through shared mechanisms, spanning the chronic cardiorenal (type 2) and renocardiac (type 4) subtypes of the cardiorenal syndrome. (**B**) The uraemic haemostatic paradox: the same patient sits high on both the thrombotic and the haemorrhagic distribution. (**C**) The four kidney-dependent judgements that a purely cardiological framing tends to collapse into one. ARN, anticoagulant-related nephropathy.

This matters for the present review because the phenomenon appears to be substantially less frequent with DOACs [45], and because it is only the acute end of a continuum: the chronic counterpart is the faster loss of GFR observed on VKAs in both trial and cohort populations (Section 6.4) [46,47,48]. Avoiding an iatrogenic, anticoagulant-driven loss of GFR is a cardiorenal benefit distinct from the avoidance of intracranial haemorrhage, and one that is not captured by the efficacy and safety endpoints of the registration trials. The cardiorenal continuum and the anticoagulation decision are shown in Figure 1.

## 4. Pharmacology of DOACs Through a Cardiorenal Lens

### 4.1. What CKD Changes

CKD alters every phase of drug disposition, not only elimination [49].

**Absorption.** Intestinal absorption of DOACs is not substantially altered by CKD in most patients. Slower and less predictable absorption has been described in patients with gastroparesis or on peritoneal dialysis, particularly for rivaroxaban, whose absorption is additionally food-dependent at the 15 and 20 mg doses.

**Distribution.** Hypoalbuminaemia is common in nephrotic syndrome and in chronic inflammation. Apixaban and rivaroxaban are highly protein-bound, so a fall in albumin increases the unbound fraction and therefore the pharmacodynamic effect at any given total concentration. The same argument applies with greater force to warfarin, which circulates more than 90% protein-bound [50]. A practical corollary is that plasma protein binding also determines dialysability: highly bound agents such as apixaban, rivaroxaban and edoxaban are poorly removed by haemodialysis, whereas dabigatran, which is only approximately 35% bound, is substantially dialysable—a property that is clinically useful in overdose and bleeding but that also makes exposure less stable in dialysis patients [51].

**Metabolism and transport.** All DOACs are substrates of P-glycoprotein (P-gp); rivaroxaban and apixaban are additionally metabolised by CYP3A4 [4,52]. Uraemia downregulates several hepatic and intestinal transporters and CYP isoforms, so drug–drug interactions may behave differently in advanced CKD from the way they behave in the populations in which they were characterised [49,52].

**Elimination.** This is the axis along which the class separates most sharply (Table 1). Approximate fractions of the absorbed dose eliminated unchanged by the kidney are 80% for dabigatran, 50% for edoxaban, 35% for rivaroxaban and 27% for apixaban [18]. This single parameter largely determines how far exposure rises as GFR falls, and therefore which agent is defensible at each CKD stage.

### 4.2. Why This Is Not Simply “Avoid the Renally Cleared Drug”

Two qualifications are needed. First, a low renal fraction reduces but does not abolish exposure–GFR dependence: apixaban exposure still rises by roughly one third in severe kidney impairment, and in haemodialysis patients steady-state concentrations with 5 mg twice daily substantially exceed those observed in patients with preserved function [57]. Second, the relationship between exposure and outcome is not symmetrical. In the ENGAGE AF-TIMI 48 population, trough concentration and anti-FXa activity were tightly correlated across doses, but rising concentration was associated with a steep increase in major bleeding and only a shallow decrease in stroke or systemic embolism, with intracranial haemorrhage essentially flat across the observed range [58]. Concretely, over a three-year horizon the modelled probability of major bleeding rose from roughly 5% at a trough concentration near zero to about 14% at 150 ng/mL, while the probability of stroke or systemic embolism fell only from about 5% to 2% across the same range [58]. In a cardiorenal population, in whom exposure drifts upward as GFR declines, this asymmetry means that unrecognised accumulation buys very little additional protection at a substantial haemorrhagic cost.

### 4.3. Dose-Reduction Rules Are Indication-Specific

The criteria summarised in Table 1 are those approved for non-valvular AF, and they do not transfer to venous thromboembolism. The distinction is easily lost, because the same tablet strengths serve both indications, but the rules governing dose reduction differ in ways that matter precisely in the patient with impaired kidney function.

Apixaban illustrates the point most sharply. In AF the dose falls to 2.5 mg twice daily when at least two of age ≥ 80 years, body weight ≤ 60 kg and serum creatinine ≥ 1.5 mg/dL are present, or when CrCl is 15–29 mL/min. In the treatment of venous thromboembolism none of these criteria applies: the regimen is 10 mg twice daily for seven days followed by 5 mg twice daily, extended prevention of recurrence is 2.5 mg twice daily after six months, and at CrCl 15–29 mL/min the label advises caution rather than a reduced dose [59]. The same elderly, low-weight patient with CKD therefore receives a reduced dose for stroke prevention and a full dose for the treatment of pulmonary embolism—counter-intuitive, but correct.

Rivaroxaban differs again. In AF, reduction to 15 mg once daily at CrCl 15–49 mL/min is the standard recommendation. In the treatment of deep vein thrombosis and pulmonary embolism, patients with CrCl 15–49 mL/min still receive 15 mg twice daily for the first three weeks, after which a reduction from 20 mg to 15 mg once daily is only to be considered where the assessed bleeding risk outweighs the risk of recurrence—a recommendation derived from pharmacokinetic modelling and explicitly not studied in that clinical setting [60]. Edoxaban and dabigatran are more straightforward, applying the same reduction criteria across indications, although both require at least five days of initial parenteral anticoagulation when used for venous thromboembolism [61,62].

Two consequences follow for the cardiorenal patient. First, a dosing rule quoted from the AF literature cannot be assumed to hold in venous thromboembolism; this applies with particular force to the discordance studies discussed in Section 7, every one of which was conducted in an AF cohort, so that their estimates of how often the choice of equation changes the dose are specific to AF labelling and should not be extrapolated. Second, the renal threshold below which each agent is not recommended is, by contrast, indication-independent: CrCl 15 mL/min for the three factor Xa inhibitors and 30 mL/min for dabigatran, in every indication.

## 5. The Individual Agents in the Cardiorenal Patient

### 5.1. Dabigatran

Dabigatran etexilate is a prodrug converted by plasma and hepatic esterases to dabigatran, a direct, reversible, competitive inhibitor of the active site of thrombin, acting on both free and fibrin-bound thrombin [51]. Oral bioavailability is low (~6.5%) and heavily P-gp dependent, which explains its susceptibility to P-gp inhibitors and inducers [63].

In RE-LY, dabigatran 150 mg twice daily was superior to warfarin for stroke and systemic embolism with similar major bleeding, whereas 110 mg twice daily was non-inferior with less major bleeding; both doses reduced intracranial haemorrhage [64]. The pre-specified renal analysis of RE-LY showed that the relative benefit of both doses was preserved across baseline kidney function, with the absolute advantage greatest at lower GFR [65]. Real-world comparative data place dabigatran close to rivaroxaban and apixaban for effectiveness in non-valvular AF (NVAF), with a more favourable major bleeding profile than rivaroxaban [66].

Dabigatran is the only DOAC with a licenced specific antidote. Idarucizumab, a humanised monoclonal antibody fragment with approximately 350-fold greater affinity for dabigatran than dabigatran has for thrombin, produces immediate and complete reversal and is given as two 2.5 g boluses no more than 15 min apart [53,54]. The comparison with vitamin K antagonism is not close: reversal is a matter of minutes rather than hours, requires no monitoring, and needs no thawing of plasma—although vitamin K and four-factor prothrombin complex concentrate remain far cheaper and universally available.

From a cardiorenal standpoint, dabigatran is the least attractive agent. Its renal dependence is the highest of the class, its exposure approximately doubles in moderate and rises further in severe impairment, and it is contraindicated in the EU below a CrCl of 30 mL/min. It is also the agent for which a signal of drug-induced kidney injury has been most examined: in a large Asian AF cohort, dabigatran was associated with a lower risk of acute kidney injury than warfarin [67], which supports the wider argument that anticoagulant choice has kidney consequences, while not making dabigatran a rational choice in established CKD.

### 5.2. Rivaroxaban

Rivaroxaban is a direct FXa inhibitor active against both free and prothrombinase-bound enzyme. Approximately one third of the dose is excreted unchanged in urine, the remainder undergoing CYP3A4- and CYP2J2-mediated metabolism [68]. ROCKET AF randomised 14,264 patients with NVAF at moderate-to-high stroke risk (median CHADS_2_ 3, median age 73) to rivaroxaban 20 mg once daily—reduced to 15 mg once daily for CrCl 30–49 mL/min—or dose-adjusted warfarin [69]. In the per-protocol as-treated population, stroke or systemic embolism occurred in 188 patients on rivaroxaban (1.7% per year) versus 241 on warfarin (2.2% per year), establishing non-inferiority (hazard ratio 0.79, 95% CI 0.66–0.96); in the intention-to-treat analysis the difference was not significant for superiority (hazard ratio 0.88, 95% CI 0.74–1.03; *p* = 0.12). Major and clinically relevant non-major bleeding did not differ overall (14.9% versus 14.5% per year), but intracranial haemorrhage (hazard ratio 0.67, 95% CI 0.47–0.93) and fatal bleeding (hazard ratio 0.50, 95% CI 0.31–0.79) were less frequent with rivaroxaban, while major gastrointestinal bleeding was more frequent (3.2% versus 2.2%; *p* < 0.001) [69]. Two features temper the comparison: time in therapeutic range in the warfarin arm averaged 55%, lower than in the other pivotal trials, and the median baseline creatinine clearance was 67 mL/min, so the trial was not powered for advanced kidney disease.

Of the FXa inhibitors, rivaroxaban carries the least favourable cardiorenal profile. Its once-daily schedule produces higher peak concentrations than twice-daily regimens; comparative real-world analyses consistently show more gastrointestinal and major bleeding than apixaban [70,71]; and the dose-discordance literature identifies rivaroxaban as the agent most sensitive to the equation used to estimate kidney function, with discordance reaching 34% in some cohorts [72]. In advanced non-dialysis CKD the same pattern holds: in a nationwide comparison restricted to stages G4–G5, rivaroxaban was associated with more major bleeding than apixaban without any gain in stroke protection [73] (Section 6.2). In haemodialysis, the Valkyrie trial compared rivaroxaban with a VKA and provides the only randomised vascular-calcification-oriented comparison in this population [35].

### 5.3. Edoxaban

Edoxaban is a once-daily direct FXa inhibitor with rapid absorption, a bioavailability of approximately 62%, dual elimination (approximately 50% renal, the remainder biliary and intestinal) and minimal CYP involvement [74,75]. Anti-FXa activity shows dose-proportional inhibition and is the pharmacodynamic marker of choice; prothrombin time and activated partial thromboplastin time are unsuitable for quantification [74].

In ENGAGE AF-TIMI 48, both edoxaban regimens were non-inferior to warfarin for stroke and systemic embolism with significantly less bleeding, including intracranial haemorrhage, and a reduction in cardiovascular death [76]. In Hokusai-VTE, edoxaban was as effective as warfarin for VTE with fewer clinically relevant bleeds. In ELDERCARE-AF, edoxaban 15 mg once daily in patients aged ≥80 years ineligible for standard anticoagulation reduced stroke and systemic embolism without a significant increase in major bleeding [77]—a regimen of direct relevance to the frail, low-weight, low-GFR cardiorenal phenotype; a dedicated analysis of dose reduction in patients aged ≥80 years supports the same conclusion [78]. Exposure data in cancer-associated and non-cancer VTE are also available [79].

Two properties define edoxaban’s cardiorenal position. First, dose reduction to 30 mg is triggered not only by CrCl 15–50 mL/min but also by body weight ≤ 60 kg or concomitant strong P-gp inhibitors—a multi-criterion rule that is frequently misapplied in exactly the elderly CKD population in which all three criteria cluster [80]. Second, the ENGAGE AF-TIMI 48 analysis of patients with CrCl > 95 mL/min showed reduced efficacy relative to warfarin, the basis of a regulatory restriction in the USA; this is the mirror image of the accumulation problem and a reminder that both tails of the GFR distribution are hazardous [76]. Experimentally, FXa inhibition by edoxaban has been reported to exert antioxidant effects in the kidney, raising the possibility of a class effect beyond anticoagulation itself [81].

### 5.4. Apixaban

Apixaban is a direct FXa inhibitor with the lowest renal dependence of the class (~27%) and the most favourable safety profile in comparative data. In ARISTOTLE it reduced stroke and systemic embolism by 21%, major bleeding by 31% and all-cause mortality by 11% relative to warfarin [82,83], and in AMPLIFY it was non-inferior to conventional therapy for VTE with substantially less bleeding [84]; real-world data are concordant [85], and its clinical pharmacokinetics are well characterised [86].

Two renal analyses are directly relevant here. By baseline kidney function, apixaban was superior to warfarin irrespective of the equation used to estimate GFR—Cockcroft–Gault, CKD-EPI or cystatin C—with the largest relative reduction in major bleeding in patients with eGFR ≤ 50 mL/min [87]. Over time, roughly one in seven patients developed worsening kidney function during follow-up, and the benefit of apixaban persisted in that subgroup [88]. A separate analysis of the dose-reduction criteria themselves showed that patients meeting only one of advanced age, low body weight or raised creatinine derived benefit from the full 5 mg twice-daily dose [89]—the clinical counterpart of the tabulating error discussed in Section 7.

These findings have made apixaban the most frequently used agent in advanced CKD, although the comparative evidence supporting that preference is observational and the choice remains a matter of clinical judgement rather than of demonstrated superiority in a randomised trial. Its pharmacokinetics have been characterised at steady state in haemodialysis patients, where 2.5 mg twice daily produces exposure comparable to that of 5 mg twice daily in patients with preserved function, while 5 mg twice daily produces substantially higher exposure [57]. Large observational analyses in dialysis populations report less major bleeding with apixaban than with warfarin, with a possible reduction in thromboembolic events at the 5 mg dose [90], and systematic reviews in stage G4–G5 CKD reach broadly concordant conclusions [91,92]; cohort data stratified by kidney function point the same way [93]. Apixaban has also been compared favourably with rivaroxaban in AF with valvular heart disease [94] and with warfarin for VTE in long-term dialysis [95].

It should be stated plainly that this evidence is predominantly observational and vulnerable to confounding by indication; the randomised trials in dialysis (RENAL-AF and AXADIA-AFNET 8) were terminated early or underpowered and did not demonstrate superiority.

### 5.5. Polypharmacy and the Second Route to Accumulation

Patients with AF and CKD are almost by definition polymedicated, and in this population drug interactions and reduced clearance act on the same target. A P-gp inhibitor raises DOAC exposure by restricting efflux; a falling GFR raises it by restricting elimination; the two combine multiplicatively in a patient who is also elderly and of low body weight. Uraemia additionally downregulates intestinal and hepatic transporters, so interaction magnitudes characterised in populations with preserved kidney function cannot simply be transferred [49,52].

The practical consequences follow. Polypharmacy does not abolish the relative advantage of DOACs over VKAs [96,97], but it shifts absolute risk: amiodarone, fluconazole, rifampicin and phenytoin have each been associated with significant changes in major bleeding [98], and P-gp– and CYP3A4-modulating drugs alter exposure in an agent-specific manner [99]. In ENGAGE AF-TIMI 48 patients receiving amiodarone derived particular benefit from the lower edoxaban dose [100], and a comparable analysis exists for apixaban [101]—in both cases the interaction was managed by reducing the dose rather than by withholding the drug. That principle generalises: in patients with AF on multiple therapies the risk of cardiovascular death is roughly 30% higher without a corresponding excess of stroke [97], which argues for reviewing the rest of the prescription rather than for withholding anticoagulation from the patient who needs it most.

## 6. DOACs Across the CKD Spectrum

### 6.1. Stages G1–G3b

In mild to moderate impairment the evidence is strong and consistent. All four agents were studied in this range in the pivotal trials, and pre-specified renal subgroup analyses show preserved or enhanced relative benefit: because absolute event rates rise as GFR falls, the absolute benefit of a DOAC over warfarin is generally larger in CKD than in patients with preserved function [102,103]. Meta-analyses restricted to CKD populations reach the same conclusion [104], and narrative syntheses addressed specifically to the AF–CKD overlap concur [105,106]. Current KDIGO guidance recommends DOACs in preference to VKAs for thromboprophylaxis in AF across CKD stages G1–G4, while acknowledging the weakness of the underlying evidence [107].

Within this range the choice between agents rests on large observational comparisons, whose populations are dominated by preserved or mildly reduced function. Marston et al. analysed 21,038 patients and found comparable effectiveness with differences in bleeding [108]; Lip et al. reported less minor gastrointestinal bleeding with apixaban than with VKAs, with rivaroxaban and dabigatran comparable [70]; and Lau et al., in 527,226 patients, found apixaban associated with a lower risk of gastrointestinal bleeding than dabigatran, edoxaban and rivaroxaban at similar effectiveness [71]. Comparable conclusions emerge from indirect comparisons in VTE [109], from apixaban-focused analyses [110] and from CKD-specific real-world cohorts [111]. Meta-analytic synthesis across the phase III trials confirms the class-level reduction in stroke, intracranial haemorrhage and mortality relative to warfarin, with heterogeneity in gastrointestinal bleeding [103].

### 6.2. Stages G4 and G5 Not on Dialysis

This is the population the review is ostensibly about, and the one for which randomised evidence is thinnest. The pivotal trials excluded patients below a creatinine clearance of 30 mL/min—25 mL/min for ARISTOTLE—so practice here rests on pharmacokinetic reasoning and observational cohorts [112,113,114,115]. Three recent analyses have nonetheless changed what can be said.

**Which agent.** In two nationwide US claims databases, Fu et al. compared new initiators of warfarin with apixaban (12,488 matched patients) and of rivaroxaban with apixaban (5720) in non-dialysis CKD stages G4–G5. Both warfarin and rivaroxaban were associated with more major bleeding than apixaban, with no difference in ischaemic stroke [73]. This is the clearest head-to-head signal available in advanced CKD, and it is concordant with the pharmacology: apixaban has the lowest renal fraction of the class and the least peak-to-trough variation.

**Which dose.** The labelling question is not academic here, because the FDA and EMA criteria diverge precisely in this range, and up to 40% of patients with CrCl below 30 mL/min receive the standard dose under FDA rules. Xu et al. followed 4313 patients with AF and CKD G4–G5 initiating apixaban, of whom 1705 received 5 mg twice daily and 2608 received 2.5 mg. The higher dose was associated with more bleeding (4.9 versus 2.9 events per 100 person-years) with no difference in stroke, systemic embolism or death [116]. The mean eGFR was identical in the two groups, at 24 mL/min/1.73 m^2^. In other words, the reduced dose bought a substantial reduction in harm at no measurable cost in efficacy—an outcome-based argument for the European criteria over the American ones, and the most concrete illustration in this review of how a labelling rule, rather than a pharmacological property, determines what happens to the patient.

**Whether at all.** The prior question—anticoagulation versus none—has never been addressed by a randomised trial in this population. Using a three-group target trial emulation across Medicare and commercial claims, Chen et al. compared apixaban and warfarin with no oral anticoagulation. Relative to non-use, apixaban increased hospitalisation for major bleeding (hazard ratio 1.32, 95% CI 1.11–1.58) and reduced ischaemic stroke (hazard ratio 0.46, 95% CI 0.32–0.66); the trade-off was more favourable for apixaban than for warfarin [117]. The net benefit is therefore real but narrow, and it is determined by where the individual patient sits on two risk distributions that, as Section 3.2 argued, are driven by shared mechanisms rather than independent ones.

Taken together, and with the proviso that none of this evidence is randomised: apixaban is the agent for which the comparative data are most favourable, the reduced dose is defensible and on current evidence probably preferable, reduced-dose edoxaban and rivaroxaban are reasonable alternatives [118,119], and dabigatran is contraindicated. These are statements about the balance of available evidence, not treatment recommendations; the differences between agents derive from non-randomised comparisons in which residual confounding and differences in baseline characteristics cannot be excluded, and in which channelling of frailer patients towards particular agents is plausible. Position papers from nephrology and thrombosis societies converge on the same position while acknowledging the weakness of the evidence [45], and KDIGO extends its DOAC preference to stage G4 [107]. What none of this resolves is the dosing question in G5 non-dialysis, where the observational cohorts thin out and the pharmacokinetic extrapolation carries most of the weight.

### 6.3. Dialysis (G5D)

Systematic reviews and meta-analyses comparing DOACs with VKAs in dialysis report less major bleeding with DOACs and no clear difference in thromboembolic events [92,120,121], and observational cohorts support apixaban specifically [90]. Against this, the two randomised trials were both underpowered and neutral: RENAL-AF stopped early after 154 of a planned 762 patients, with no significant difference in major or clinically relevant non-major bleeding between apixaban and warfarin [122], and AXADIA-AFNET 8 randomised 97 patients to apixaban 2.5 mg twice daily or phenprocoumon without demonstrating superiority on either safety or efficacy [123]. The benefit of any anticoagulation for AF in dialysis has itself been questioned [124,125,126], and residual confounding in observational designs is substantial. The honest summary is that the randomised evidence in G5D is not negative but absent, and that the observational signal favouring apixaban should be read accordingly. Guideline positions in this population remain deliberately cautious [36].

In practice, the cardiorenal decision in G5D is less “which DOAC” than “whether to anticoagulate at all”, and it should be individualised against competing risks, dialysis access, fall risk and patient priorities, including quality of life [127]. Analogous uncertainty applies to procedural settings such as transcatheter aortic valve replacement in CKD [128], and pharmacokinetic data during continuous renal replacement therapy remain limited to single agents [129].

In a retrospective cohort from Taipei Veterans General Hospital comparing DOACs with warfarin in AF with advanced kidney disease, DOACs were associated with a lower risk of stroke or systemic embolism (adjusted hazard ratio 0.29) and of overall ischaemic events (adjusted hazard ratio 0.42), with a similar or slightly lower bleeding risk; apixaban performed best on both axes [130].

### 6.4. The Kidney as an Outcome of Anticoagulation

A specifically cardiorenal question is whether the choice of anticoagulant alters the trajectory of kidney function itself. The evidence now converges from four directions.

In RE-LY, GFR declined in all arms over a mean of 30 months, but the fall was significantly greater with warfarin than with either dabigatran dose, and the difference was amplified in patients with diabetes and in those with prior VKA exposure [46]. In ROCKET AF, the proportion of patients meeting a ≥20% fall in CrCl did not differ between arms, although mean CrCl declined slightly more with rivaroxaban [131]—a discordance with RE-LY that has not been resolved and that may reflect differences in definition and in creatinine sampling rather than in drug effect. In ARISTOTLE, worsening kidney function occurred in approximately 14% of patients over a year and was not clearly influenced by treatment assignment, while the efficacy and safety advantages of apixaban persisted in that subgroup [88].

Observational data are more consistent. In a large anticoagulated AF cohort, warfarin was associated with higher risks of a ≥30% decline in eGFR, of doubling of serum creatinine and of acute kidney injury than DOACs [47], findings echoed in European cohorts [48] and in CKD populations exposed to warfarin [43]. In an Asian AF cohort, dabigatran was associated with a lower incidence of acute kidney injury than warfarin [67]. Analyses framed explicitly as cardiorenal have compared the two classes on combined cardiac and kidney endpoints [21].

Two cautions apply. The trial analyses are post hoc, use creatinine-based estimates collected at irregular intervals, and define “worsening kidney function” heterogeneously; observational comparisons are exposed to confounding by indication, since patients started on warfarin differ systematically from those started on a DOAC. The claim that DOACs are renoprotective is therefore not established. Patients commenced on a VKA differ systematically from those commenced on a DOAC in age, comorbidity, frailty and in the reasons for which one agent was chosen over the other, and no degree of statistical adjustment removes that difference; the observed association between anticoagulant class and the rate of GFR decline should accordingly be read as hypothesis-generating. What is defensible—and what a cardiorenal review should say—is that kidney function decline differs by anticoagulant class in a direction consistently unfavourable to VKAs, that this endpoint has never been a pre-specified as primary endpoint in any trial, and that it should be.

## 7. Estimating Kidney Function for Dose Selection: A Metrological Problem with Clinical Consequences

### 7.1. The Mismatch

Dose thresholds for DOACs are expressed in CG-estimated CrCl, because that is what the registration trials used [23,24]. ROCKET AF states this explicitly: creatinine clearance was calculated with the Cockcroft–Gault formula, and the 15 mg dose-reduction threshold of 30–49 mL/min was defined on that basis [69]. Every subsequent dosing decision in routine care inherits that definition. Contemporary nephrology stages CKD using eGFR from CKD-EPI, and increasingly from the EKFC equation [132]—historically from MDRD [133]—standardised to 1.73 m^2^ of body surface area. These are different quantities, not merely different formulae: CrCl exceeds GFR because of tubular creatinine secretion, and indexed eGFR is not the same as the absolute clearance relevant to drug elimination [19]. Converting an indexed eGFR to an absolute value (mL/min) before applying a dosing threshold is a necessary but frequently omitted step.

The CG equation carries specific biases. It incorporates total body weight without distinguishing lean from fat mass, and therefore overestimates CrCl in obesity [19]; conversely, it weights age heavily, and therefore returns systematically lower values than eGFR equations in older patients [134]. CKD-EPI and MDRD, for their part, overestimate measured CrCl in older individuals [134,135]. There is no configuration in which one equation is simply “correct”.

Two further developments bear directly on the dosing problem and are absent from all of the discordance studies reviewed below. The EKFC equation, built on a rescaled creatinine ratio with an age-dependent term, shows lower bias and better precision than CKD-EPI across the full age spectrum, and specifically in older adults—the population in which CG and CKD-EPI diverge most [132]. A cystatin C-based EKFC equation, requiring neither race nor sex, performs comparably and is not confounded by muscle mass [136]; a European appraisal of the competing equations sets out the practical choice [137]. Since the cardiorenal patient is frequently sarcopenic, creatinine-based estimates in this group carry a systematic upward bias that no reweighting of the same analyte can remove. Whether cystatin C-based or combined estimates would change DOAC dose selection, and in which direction, is unknown and testable.

### 7.2. What the Discordance Studies Show

Several cohorts have quantified how often the equation changes the dose.

Chan et al. studied 39,239 patients stratified by eGFR (<15, 15–50, >50 mL/min) and reported overestimation of kidney function in 21% of patients with MDRD and 17% with CKD-EPI relative to CG, against underestimation in only 2% and 1% respectively [138]. Because overestimation leads to a higher dose, the asymmetry matters clinically.

Manzano-Fernández et al. analysed 910 patients with AF and an indication for anticoagulation, and reported discordance rates that rise monotonically with age and with the severity of kidney impairment (Table 2) [139]. In the cohort as a whole, discordance relative to CG was 11.4% (MDRD) and 10.0% (CKD-EPI) for dabigatran and 10.0% and 8.5% for rivaroxaban; among elderly patients and those with CG-estimated CrCl < 60 mL/min it reached 13.2–30.4%. Apixaban was consistently least affected, remaining below 5% in every stratum, because its dose-reduction rule depends on a composite of age, weight and creatinine rather than on a single clearance threshold.

The first two rows explain the rest of the table. Where the dose depends on a single creatinine-derived threshold, changing the equation moves patients across it; where the rule is a composite in which creatinine is one of three criteria, it does not. Apixaban’s insensitivity is therefore a property of its labelling, not of its pharmacology—a point with a direct clinical counterpart, since patients meeting only one of the three criteria benefit from the full dose [89].

Pérez Cabeza et al. studied 454 patients with NVAF and found rivaroxaban most affected (34% discordance) and apixaban least (4.8%) [72]. They attributed the pattern to the heavier age weighting of CG, which yields lower values in older patients with CKD and therefore more frequent dose reduction. This differs from Manzano-Fernández et al., who identified dabigatran as most discordant; the discrepancy is explained by the treatment of the non-renal dose-reduction criteria, which were not applied uniformly across the two analyses [72,139].

Bhalodia et al. took a different approach, examining outcomes rather than concordance alone, in 1616 patients with AF receiving rivaroxaban or dabigatran at a single US centre between 2010 and 2016 [140]. Their design is worth stating precisely, because it determines what the numbers mean: among patients whose dose was correct under CG labelling, a dose was counted as discordant if the dose calculated from CKD-EPI would have differed. On that definition, discordance was infrequent—49 of 644 patients (8%) for rivaroxaban and 17 of 590 (3%) for dabigatran.

Infrequent did not mean harmless. Discordance in rivaroxaban dosing was associated with a nearly three-fold increase in thromboembolism (odds ratio 2.83, 95% CI 1.02–7.79; *p* = 0.045), with no corresponding signal for dabigatran [140].

The direction matters: because CKD-EPI tends to return higher values than CG in this population, the discordant patients were those who would have received a higher dose under CKD-EPI, and they were the ones who suffered thromboembolic events—that is, CG-based dosing had under-treated them.

Finally, Andrade et al. showed that the consequences of inappropriate dosing are asymmetrical and clinically material: overtreatment increased bleeding without reducing stroke, whereas undertreatment increased stroke without reducing bleeding; correctly dosed patients with moderate impairment had outcomes comparable to those without impairment [141]. They proposed that estimated CrCl should be calculated in all patients with clearance below approximately 70 mL/min before starting edoxaban, dabigatran or rivaroxaban. That analysis was limited by missing body weight in 36% of the cohort, which precluded assessment of dosing appropriateness in a substantial minority [141].

Similar variability in real-world dose selection has been documented across European and international practice [142,143], and concordance analyses between MDRD, CKD-EPI and CG confirm that the phenomenon is general rather than cohort-specific [144].

### 7.3. Synthesis

Three conclusions follow. First, the direction of error is not random: relative to CG, eGFR equations overestimate function in older patients with reduced GFR, and therefore favour overdosing in precisely the population at highest bleeding risk [134,135,138]. Second, the magnitude of the problem is agent-dependent, largest for rivaroxaban and dabigatran and smallest for apixaban [72,139]. Third, CG is not exempt: it overestimates clearance in obesity and in patients with preserved function [19].

The practical implication is that the equation should be treated as part of the prescription. Until outcome-anchored evidence exists for eGFR-based dosing, CrCl by CG—computed with a defensible weight descriptor and de-indexed comparisons where eGFR is used—should remain the operative quantity for dose selection, while CKD-EPI or EKFC remain the correct tools for staging, prognosis and nephrology referral [24,26].

## 8. A Practical Summary Across Stages of Chronic Kidney Disease

**Figure 2:**
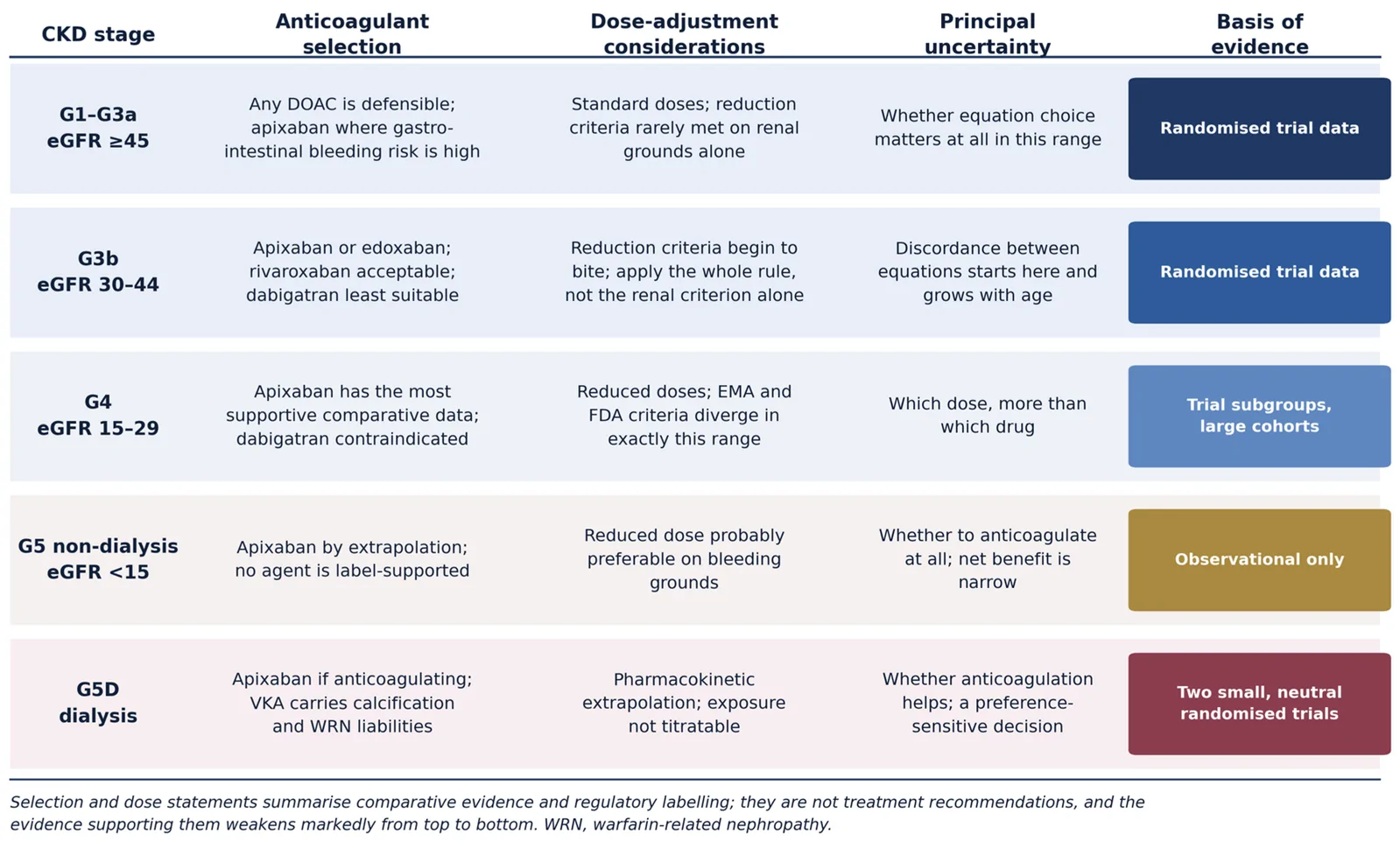
Anticoagulant selection, dose-adjustment considerations and principal areas of uncertainty across stages of chronic kidney disease, with the basis of evidence for each stratum. CG, Cockcroft–Gault; WRN, warfarin-related nephropathy.

The preceding sections can be condensed into a stage-stratified summary (Figure 2). Two cautions govern how it should be read. The statements on agent selection summarise comparative findings, predominantly observational beyond stage G3b, together with regulatory labelling; they are not treatment recommendations, and none of them substitutes for an individual assessment of thromboembolic and bleeding risk. The strength of the evidence behind them also falls steeply as GFR declines, which is why the basis of evidence is shown explicitly for each stratum rather than left to be inferred.

Four points apply across every stage. Kidney function should be staged with CKD-EPI or EKFC, and with cystatin C where muscle mass is atypical, while the dose is selected from CG-estimated creatinine clearance, de-indexed where it has been derived from an indexed eGFR; the two quantities answer different questions and should not be substituted for one another. The whole dose-reduction rule should be applied and not only its renal criterion, since age, body weight, interacting drugs and, for apixaban, serum creatinine all enter the decision [80]. Kidney function should be reassessed on a defined schedule, at least every six months when eGFR is stable and more frequently when it is not. And an unexplained rise in creatinine in an over-anticoagulated patient should prompt consideration of anticoagulant-related nephropathy before the change is attributed to progression of CKD [20,44].

Where the evidence is weakest—stage G5, whether or not the patient is receiving dialysis—the decision becomes preference-sensitive rather than protocol-driven, and is best taken jointly by a nephrologist and a cardiologist with the patient [22,36,145].

## 9. Beyond the Current Class: Factor XI Inhibition in the Cardiorenal Patient

Every anticoagulant discussed so far shares a structural limitation. Each inhibits a step that is required both for pathological thrombus formation and for normal haemostasis, so that efficacy and bleeding move together; in the cardiorenal patient, who occupies a high decile of both distributions, that coupling is what makes the therapeutic window narrow. Factor XI inhibition is of interest because it offers the prospect of loosening it.

The rationale is partly genetic. Congenital factor XI deficiency is associated with a reduced incidence of ischaemic stroke and venous thromboembolism but with little spontaneous bleeding, since factor XI amplifies thrombin generation through the contact pathway while contributing comparatively little to the haemostatic plug formed after tissue injury [146]. Pharmacological inhibition might therefore attenuate thrombosis with a smaller haemostatic penalty—the precise trade-off that constrains anticoagulation as GFR falls.

The clinical evidence is so far encouraging on safety and unresolved on efficacy. In AZALEA-TIMI 71, a phase 2b randomised trial in patients with AF, the monoclonal antibody abelacimab was compared with rivaroxaban and was stopped early by the data monitoring committee because of a marked reduction in bleeding; the trial was not powered for stroke prevention [147]. A pre-specified analysis stratified by kidney function reported that the reduction in bleeding was consistent across categories of estimated GFR, including the lower strata in which bleeding risk is greatest [148]. Against this, OCEANIC-AF, which compared the small-molecule factor XIa inhibitor asundexian with apixaban, was terminated early for inferior efficacy [149], and phase 3 programmes with abelacimab and milvexian remain in progress. The class therefore cannot yet be described as an established alternative, and the divergence between agents suggests that the degree and the mechanism of inhibition matter.

Three observations are specific to the cardiorenal setting. First, abelacimab is a monoclonal antibody cleared by reticuloendothelial catabolism rather than by the kidney, so the dosing problem that occupies much of this review—the dependence of exposure on an estimated clearance, and on which equation produced it—would not arise in the same form. Second, that advantage carries its own cost: a long half-life and monthly administration leave no scope for dose titration, and no specific reversal agent is available, in a population that undergoes frequent vascular access procedures, biopsies and unplanned surgery. Third, patients with advanced CKD have been underrepresented in the trials conducted to date, and those on dialysis have been excluded altogether, so the population in which the haemostatic argument is strongest is also the one in which the evidence is thinnest. Whether factor XI inhibition resolves the cardiorenal dilemma or merely relocates it is an empirical question that the current trial programme, as designed, will not fully answer.

## 10. Limitations

This is a narrative and not a systematic review; selection of sources was not protocol-driven, and the synthesis is interpretive. Restriction to English-language reports introduces a recognised language bias, and the purposive selection of sources means that the balance of evidence presented reflects the authors’ judgement of relevance as well as the state of the literature. Almost all of the studies discussed for advanced CKD are retrospective and subject to confounding by indication, and most treat CG as the reference standard for kidney function—a convention that reflects regulatory history rather than physiological accuracy, and one that this review has argued should be made explicit rather than assumed. Evidence in paediatric and in cancer-associated thrombosis populations, although relevant to the wider DOAC literature [150,151,152,153,154,155,156], lies outside the cardiorenal scope adopted here.

Four priorities follow for the cardiorenal community. First, prospective validation of eGFR-based dose selection against clinical outcomes, rather than against CG-derived concordance [26]. Second, adequately powered randomised trials in G4–G5D, with kidney function decline as a pre-specified outcome and not only as a safety signal; the premature termination of RENAL-AF and the small size of AXADIA-AFNET 8 [122,123] show that this will require dedicated funding and recruitment strategies rather than opportunistic sub-studies. Third, systematic incorporation of cystatin C-based and EKFC-based estimates into dosing studies [132,136,137], given the prevalence of sarcopenia in this population. Fourth, prospective characterisation of anticoagulant-related nephropathy across the DOAC class, using standardised definitions. Several of these priorities have been articulated previously from the haemostasis perspective [157]; what the cardiorenal framing adds is the requirement that kidney endpoints be pre-specified rather than reported as adverse events.

## 11. Conclusions

In the cardiorenal patient, anticoagulation is not a cardiological decision with a renal adjustment; it is a shared decision in which the kidney determines drug exposure, modifies bleeding risk, and is itself affected by the choice made. Read in that light, the evidence supports DOACs over VKAs across most of the CKD spectrum—not only because they bleed less intracranially, but because they avoid the vitamin K-dependent vascular calcification pathway and appear substantially less likely to cause anticoagulant-related nephropathy.

The open problem is metrological. Every study reviewed here treats CG-estimated CrCl as the operative quantity for dosing, while the same patients are staged with eGFR equations that systematically disagree with it in the old and in the sarcopenic. The disagreement is not symmetrical and not clinically neutral: it shifts patients across dosing thresholds in the direction of overdosing precisely where the bleeding risk is greatest. What is needed is not a further demonstration that the equations disagree—that is now established—but prospective, outcome-anchored evidence on which quantity should govern the dose. Until that evidence exists, the equation used should be recorded alongside the dose, and the choice of both should be made deliberately.

## Figures and Tables

**Table 1:** Renal handling and dose selection across the class (EU labelling). Doses and reduction criteria are those approved for non-valvular atrial fibrillation; for venous thromboembolism see Section 4.3.

Property	Dabigatran	Rivaroxaban	Edoxaban	Apixaban
Target	Thrombin (FIIa)	FXa	FXa	FXa
Oral bioavailability	~6.5% (P-gp substrate)	80–100% (with food, 15/20 mg)	~62%	~50%
Renal elimination of absorbed drug	~80%	~35%	~50%	~27%
Plasma protein binding	~35%	~92–95%	~55%	~87%
Removed by haemodialysis	Yes (~50–60% over 4 h)	No	No	No
Specific reversal agent	Idarucizumab [53,54]	Andexanet alfa [55]	Not indicated; andexanet alfa is not licenced for edoxaban reversal [55,56]	Andexanet alfa [55]
Interaction profile	P-gp only	P-gp + CYP3A4	P-gp only	P-gp + CYP3A4
Standard dose	150 mg twice daily	20 mg once daily	60 mg once daily	5 mg twice daily
Reduced dose	110 mg twice daily	15 mg once daily	30 mg once daily	2.5 mg twice daily
Criteria for reduction	Recommended: age ≥ 80 y, or concomitant verapamil. To be considered on individual assessment: CrCl 30–50 mL/min, age 75–80 y, gastritis, oesophagitis or gastro-oesophageal reflux, or otherwise increased bleeding risk	CrCl 15–49 mL/min	CrCl 15–50 mL/min, **or** weight ≤ 60 kg, **or** concomitant ciclosporin, dronedarone, erythromycin or ketoconazole. Amiodarone, quinidine and verapamil do not require dose reduction	CrCl 15–29 mL/min, **or** ≥2 of: age ≥ 80 y, weight ≤ 60 kg, creatinine ≥ 1.5 mg/dL
Threshold for non-use	Contraindicated if CrCl < 30 mL/min	Not recommended if CrCl < 15 mL/min	Not recommended if CrCl < 15 mL/min, in end-stage renal disease or on dialysis	Not recommended if CrCl < 15 mL/min or on dialysis

**Table 2:** How far the dose-reduction rule depends on the equation used.

	Dabigatran	Rivaroxaban	Apixaban
**Structure of the dose-reduction rule**	Single clearance threshold, plus discretionary clinical factors	Single clearance threshold	Single clearance threshold **or** a two-of-three composite (age, weight, creatinine)
**Does the rule depend on creatinine alone?**	Yes	Yes	No
Discordance vs. CG, overall cohort—MDRD/CKD-EPI (%) [139]	11.4/10.0	10.0/8.5	1.4/1.5
Discordance vs. CG, elderly—MDRD/CKD-EPI (%) [139]	18.3/15.1	16.2/13.2	2.1/1.9
Discordance vs. CG, CrCl < 60 mL/min—MDRD/CKD-EPI (%) [139]	30.4/26.5	26.5/22.4	3.8/4.1
Discordance in a second cohort, among correctly CG-dosed patients (%) [140]	3 (17/590)	8 (49/644)	Not studied
Clinical signal associated with discordance [140]	None detected	Thromboembolism, odds ratio 2.83 (1.02–7.79)	Not studied

## Data Availability

No new data were created or analysed in this study. Data sharing is not applicable to this article.
